# A case report of chromosome 17q22‐qter trisomy with distinct clinical presentation and review of the literature

**DOI:** 10.1002/ccr3.1298

**Published:** 2018-02-14

**Authors:** Jariya Upadia, Joseph B. Philips, Nathaniel H. Robin, Edward J. Lose, Fady M. Mikhail

**Affiliations:** ^1^ Department of Genetics University of Alabama at Birmingham Birmingham Alabama USA; ^2^ Department of Pediatrics University of Alabama at Birmingham Birmingham Alabama USA

**Keywords:** Terminal 17q trisomy, unbalanced chromosomal translocation

## Abstract

Terminal 17q trisomy is very rare but a recognizable genetic syndrome. The majority of cases reported are inherited from a balanced translocation carrier. This syndrome involves many organs and the severity ranges from mild to severe depending on the size of the 17q gain.

## Introduction

Trisomy of the distal portion of long (q) arm of chromosome 17 is a rare but recognized syndrome, with approximately 20 similar cases reported to date. The common characteristic features reported among individuals with distal 17q trisomy overlap, and include microcephaly, short stature, distinct facial features, short and webbed neck, low posterior hairline, rhizomelia, polydactyly, syndactyly, and others 1, 2, 3, 4. Both de novo and inherited cases have been reported 5, 6.

Here, we report an infant female with a 24.3 Mb terminal 17q trisomy (17q22–17qter) as a result of an unbalanced translocation between the q arms of chromosomes 10 (break point at 10qter) and 17 (break point at 17q22). This case is unique because the patient has an isolated terminal 17q trisomy and presents with pleural effusion and ascites, which have not been previously reported in the literature as part of the phenotype. We review and compare this case with previously reported cases with distal 17q trisomy, including the size and location of the 17q gain.

## Clinical Report

The proband is a Caucasian female born to unrelated healthy parents: a 21‐year‐old mother and a 38‐year‐old father. The family history was unremarkable. The pregnancy was complicated by polyhydramnios beginning at 15 weeks. A subsequent ultrasound identified bilateral pleural effusions, shortened long bones, and cleft lip.

**Figure 1:**
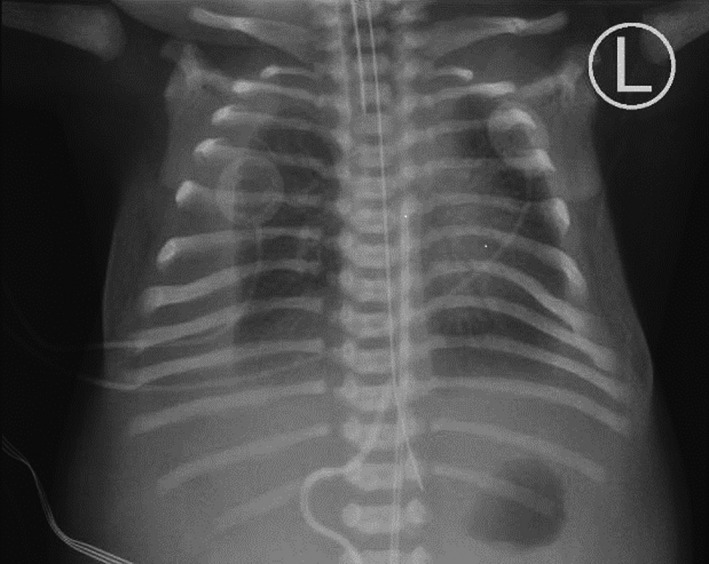
Chest X‐ray AP supine showed moderate right‐side pleural effusion and fluid in the left costophrenic angle region.

The infant was born by an unremarkable vaginal delivery at 37 weeks. Birthweight was 2900 g (38th centile), length was 48 cm (32nd centile), and head circumference 33.5 cm (41st centile). Apgar scores were 2, 5, and 7 at one, five, and ten minutes, respectively. The infant was hypotonic and there was respiratory distress. She was intubated and transferred to the NICU. On physical examination, it was noted that she had bitemporal narrowing, frontal bossing, short neck with low posterior hairline, short palpebral fissures, hypertelorism, broad nasal bridge, low‐set and posteriorly rotated ears, thickened helix, micrognathia, and right unilateral cleft lip and complete cleft palate. Tone was symmetrically depressed in all limbs, and deep tendon reflexes were present. Chest X‐ray at that time showed fluid within the right pleural space with volume loss of the right lung, and fluid–atelectasis in the left costophrenic angle region (Fig. 1). Cerebral ultrasound revealed no structural defects and small incidental cysts in the choroid plexus bilaterally. Abdominal ultrasound showed small amount of ascites and normal liver, gall bladder, pancreas, and kidneys. Echocardiography demonstrated a patent ductus arteriosus with small left to right shunt. Pleural effusions resolved around 1 week after birth.

**Figure 2:**
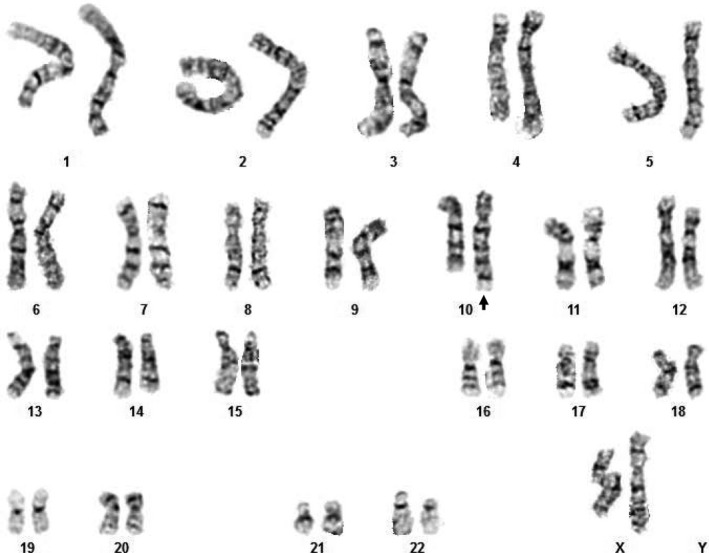
G‐banded chromosome analysis of the cultured amniotic fluid cells. The karyotype demonstrated an unbalanced 10q;17q translocation with the formation of a derivative chromosome 10. Note the terminal 17q trisomy (17q22–17qter) at the bottom of the derivative chromosome 10 (arrow).

**Figure 3:**
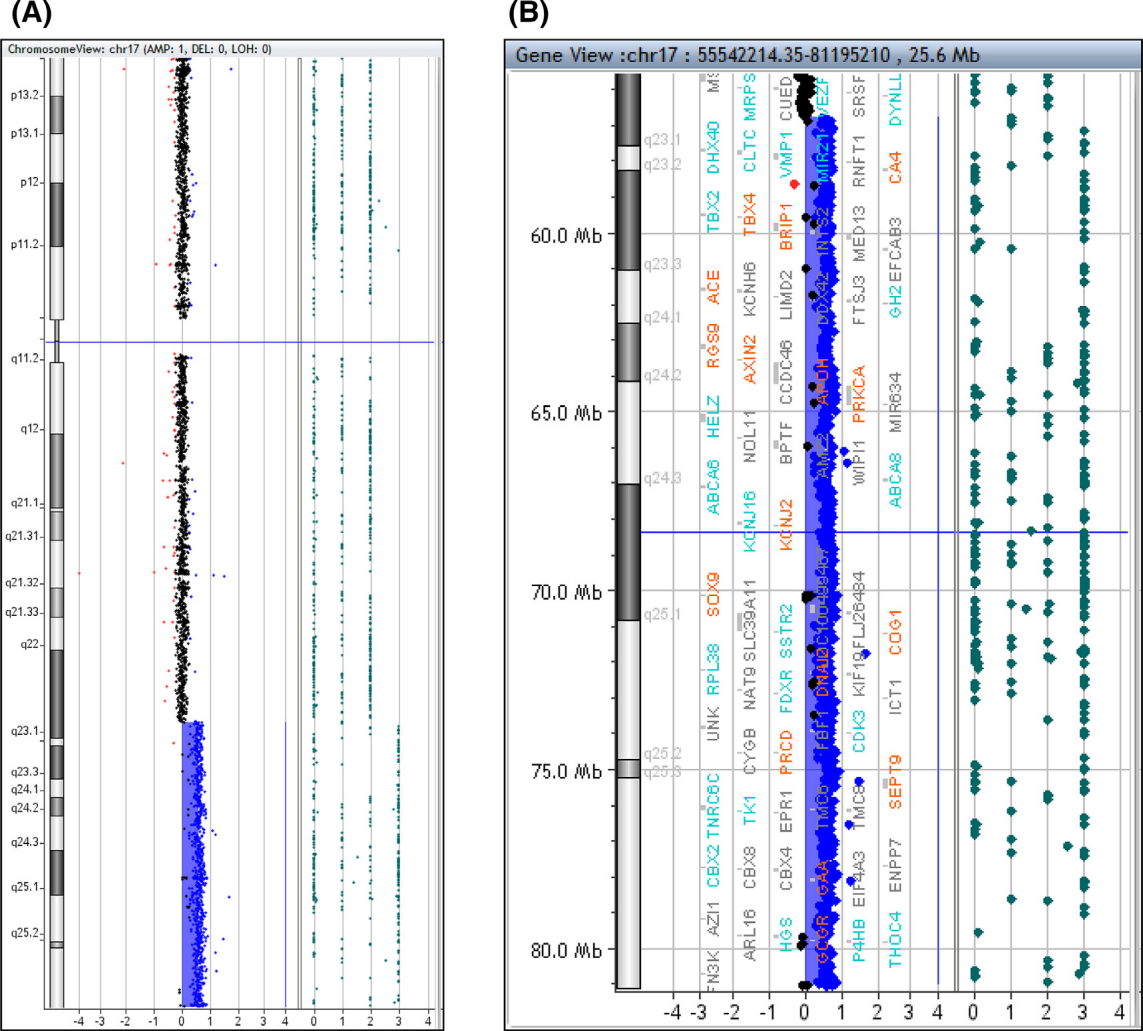
Array comparative hybridization (aCGH) plots of chromosome 17. Note the approximately 24.3 Mb terminal 17q one‐copy gain. (A) Whole chromosome 17 view; (B) Terminal 17q zoomed in view.

Cytogenetic studies included G‐banded chromosome analysis of cells derived from amniotic fluid culture performed at 18 weeks of gestation, which demonstrated an unbalanced translocation involving the q arms of chromosomes 10 and 17 with break points at bands 10q26.3 and 17q22, respectively, resulting in the formation of a derivative chromosome 10. The karyotype of the patient was reported as 46,XX,der(10)t(10;17)(q26.3;q22) (Fig. 2). Array comparative genomic hybridization (aCGH) analysis using the Agilent 4 × 180k aCGH+SNP array performed on DNA from the cultured amniotic fluid cells demonstrated a one‐copy gain (trisomy) of the terminal ~24.3 Mb of 17q with a break point at band 17q22 (hg19 break point at linear genomic position 56,767,827 bp) (Fig. 3). This duplication encompasses more than 200 annotated RefSeq genes. Array comparative genomic hybridization analysis did not demonstrate a terminal 10q loss, which suggests that the 10q break point is very terminal in the telomere or telomere‐associated repeats. Metaphase fluorescence in situ hybridization (FISH) analysis using the 10q and 17q subtelomere probes (Abbott) confirmed these results. Cytogenetic studies of the proband's parents were not performed.

## Discussion

Trisomy of the distal portion of chromosome 17q arm is a rare and recognizable genetic syndrome. It was first described in 1978 by Berberich et al. who reported three cousins with 17q23‐qter trisomy due to the unbalanced segregation of a familial balanced translocation. They were found to have similar features and organ anomalies. Now, with over 20 cases reported, the phenotype of the distal 17q trisomy syndrome consists of microcephaly, facial features including short palpebral fissures, down‐slanting palpebral fissures, hypertelorism, flattened and broad nasal bridge, wide mouth, thin upper lip, downturned corner of the mouth, low‐set and posterior rotated ears, micrognathia, long and smooth philtrum, cleft lip and/or palate, joint laxity and other organ anomalies such as cardiac defects, renal, and limb anomalies 2, 5, 7, 8. De novo and mosaic cases have been reported 5, 9, 10, 11. The majority of cases are due to unbalanced translocations inherited from balanced translocation carriers 12, 13. One reported case is due to an inverted duplication of the 17q24‐q25.3 region 6.

While the phenotype of most cases is complicated by terminal monosomy of the other chromosomes involved in the unbalanced translocation, cases with isolated distal 17q trisomy have been reported including our case [9; Kelly et al., 2002]. Interestingly, the phenotype of distal 17q trisomy is relatively consistent with or without terminal monosomy of a part of another chromosome, suggesting that the distal 17q trisomy is the main determinant of these features.

In approximately 85% of the cases, the distal 17q trisomy is inherited from a balanced carrier parent. Among these cases, the majority had normal birthweight with an average birthweight of 2913 g. Individuals with distal trisomy of chromosome 17q have a range of findings, which are summarized in Table 1. The percentages included are only for those cases in which the finding was specifically described as present or absent.

**Table 1:** Summary of clinical characteristics in distal 17q trisomy categorized by frequency

Frequency (%)	Characteristic features	Present case
75–100	Developmental delay (20 of 20)	+
	Short stature (20 of 22)	+
	Microcephaly (14 of 18)	−
	Wide mouth with thin upper lip (15 of 17)	+
	Flattened nasal bridge (12 of 16)	+
	Low‐set and posterior rotated ears (16 of 21)	+
	Widely spaced nipple (nine of 12)	+
	Bitemporal narrowing (12 of 16)	+
50 to <75	Cryptorchidism (seven of 10)	NA
	Short neck (12 of 17)	+
	Frontal bossing (13 of 19)	+
	Low posterior hairline (12 of 17)	+
	Hypertelorism (10 of 16)	+
	Brain anomaly (10 of 16)	+
	Long and smooth philtrum (five of eight)	−
	Facial asymmetry (six of 10)	−
	Micrognathia (10 of 16)	+
	Downturn corner of the mouth (10 of 19)	+
	High arch palate (10 of 20)	−
	Webbed neck (nine of 18)	−
	Adductus deformity of the thumb (six of 11)	−
	Heart defect (seven of 12)	−
	Widow peak (eight of 16)	+
25 to <50	Short palpebral fissures (four of 10)	+
	Down‐slanting palpebral fissures (seven of 16)	+
	Preauricular pit (three of seven)	−
	Rhizomelia (eight of 19)	−
	Beaked nose (two of seven)	−
	Scoliosis (four of 11)	−
	Cleft lip and/or cleft palate (seven of 19)	+
	Postaxial polydactyly (seven of 21)	−
	Long and thin fingers (four of 13)	−
<25	Overlapped toes (three of 13)	−
	Syndactyly (four of 18)	−
	Eye abnormalities (optic nerve hypoplasia, nystagmus, strabismus, lateral rectus palsy)	−
	Large anterior fontanel	+
	Abnormal genitalia (small testes, bifid scrotum, penile chordee)	−
	Inguinal hernia	−
	Short metacarpal bones	−
	Sensorineural hearing loss	−
	Hirsutism	+
	Small hands and feet	−
	Kidney anomaly	−
	Ascites	+
	Pleural effusion	+

NA, not applicable; +, feature present; −, feature absent. Babovic‐Vuksanovic et al. 5; Berberich et al. 1; Kelly et al. 14; Bridge et al. 7; Caine et al. 8; Fryns et al. 9; Gallien et al. 2; Naccache et al. 12; Ohdo et al. 3; Orye et al. (1985); Robb et al. (1987); Serokin et al. 11; Shimizu et al. 6; Turleau et al. 4; Yamamoto et al. 15.

This syndrome involves many organs. A congenital heart defect is present in 50% of affected individuals including pulmonary stenosis, atrial septal defect, subaortic stenosis, patent ductus arteriosus, and Tetralogy of Fallot [Berberich et al., 1978; 2, 7, 8]. Eye abnormalities have been reported include exotropia, optic nerve lesion, strabismus, nystagmus, lateral rectus palsy, disconjugate gaze, and optic nerve hypoplasia 3, 5, 7, 14. Kidney anomalies were reported in two cases, which include duplex kidney, and absent/hypoplastic kidney 1, 2, 7, 14. Brain anomalies have been reported in approximately 62% of cases with no clinical details provided from the previous studies except for two unrelated patients who were reported to have cerebral atrophy 1, 3, 7, 12, 15. Other rare findings reported include anemia 8, sensorineural hearing loss 10, short metacarpal bone 5, craniosynostosis 5, inguinal hernia 11, bone dysplasia 13, cavernous hemangioma 7, small hands and feet [Shimizu et al., 1988], and hirsutism 6. Hydrop fetalis was also reported in a female infant with distal trisomy 17q (q23‐qter), but this patient also has a deletion of the terminal region of chromosome 12p (p13.3‐pter) 8. Many of the reported clinical features overlap with other genetic syndromes such as 22q11.2 deletion syndrome, Trisomy 13, Tetrasomy 18p, Trisomy 21, chromosomal deletions, and peroxisomal disorders.

Our case has typical features of distal 17q trisomy as shown in Table 1. This is the first terminal 17q trisomy case, in which the break point was precisely mapped by aCGH. And is the first case reported to date with terminal 17q gain to present with pleural effusion and ascites. Although the etiology of pleural effusion and ascites in our case is unknown, these findings have been reported in other genetic syndromes such as trisomy 21 16, 17, 18, Turner syndrome 19, Noonan syndrome 20, 21 and chromosomal deletions 22, 23, 24. We suggest that the spectrum of phenotypes expressed by patients with 17p trisomy includes hydrops fetalis.

## Authorship

JU: was the primary Genetics physician for this patient, leader of the evaluation of the patient, and the primary author of the manuscript. NHR: was the attending Genetics physician who saw the patient initially, assisted in the writing of the manuscript. JBP: was the attending neonatologist for this patient, assisted in writing the manuscript particularly providing additional clinical details. EJL: was the attending Genetics physician for this patient, involved in the clinical care and evaluation. FMM: was the cytogeneticist who assisted in writing the manuscript particularly providing cytogenetic study details.

## Conflict of Interest

None declared.
